# Endoscopic management of pancreatic duct stones and stricture in chronic pancreatitis using a fully covered self-expandable metal stent

**DOI:** 10.1055/a-2966-7129

**Published:** 2026-10-08

**Authors:** Nan Zeng, Beibei Li, Jingbo Yang

**Affiliations:** 1 Department of Gastroenterology & Gastrointestinal Endoscopy Center 499778 Shenzhen University First Affiliated HospitalShenzhenGuangdong ProvinceChina

A main pancreatic duct stricture on the papillary side of a pancreatic duct stone may impede device passage and stone extraction. 1​ We temporarily placed a fully covered self-expandable metal stent (FCSEMS) to dilate the stricture and facilitate balloon extraction without lithotripsy. 2​

A 33-year-old woman with a 10-year history of recurrent abdominal pain and multiple admissions for acute pancreatitis was referred to our institution. Magnetic resonance cholangiopancreatography showed pancreatic atrophy, main pancreatic duct dilation, and an intraductal filling defect suggestive of a stone. Endoscopic ultrasonography showed a 2.3×3.5-mm hyperechoic intraductal focus without acoustic shadowing.

Pancreatography showed a tortuous main pancreatic duct approximately 6 mm in diameter, a 5×6-mm filling defect, and a stricture on the papillary side of the stone ( Fig. 1 ). Given the duct tortuosity and tight stricture, direct stone extraction was considered unlikely to succeed. As extracorporeal shock wave lithotripsy and other lithotripsy modalities were unavailable, temporary FCSEMS placement was selected to facilitate subsequent stone extraction, taking procedural safety and cost into consideration. After sequential bougie dilation (7-Fr, 8.5-Fr, and 9-Fr), a 10×60-mm FCSEMS was deployed across the stricture ( Video 1 ). A 7-Fr×7-cm biliary plastic stent was placed prophylactically ( Fig. 2 ). Whitish stone debris was observed after FCSEMS deployment.

**Fig. 1:**
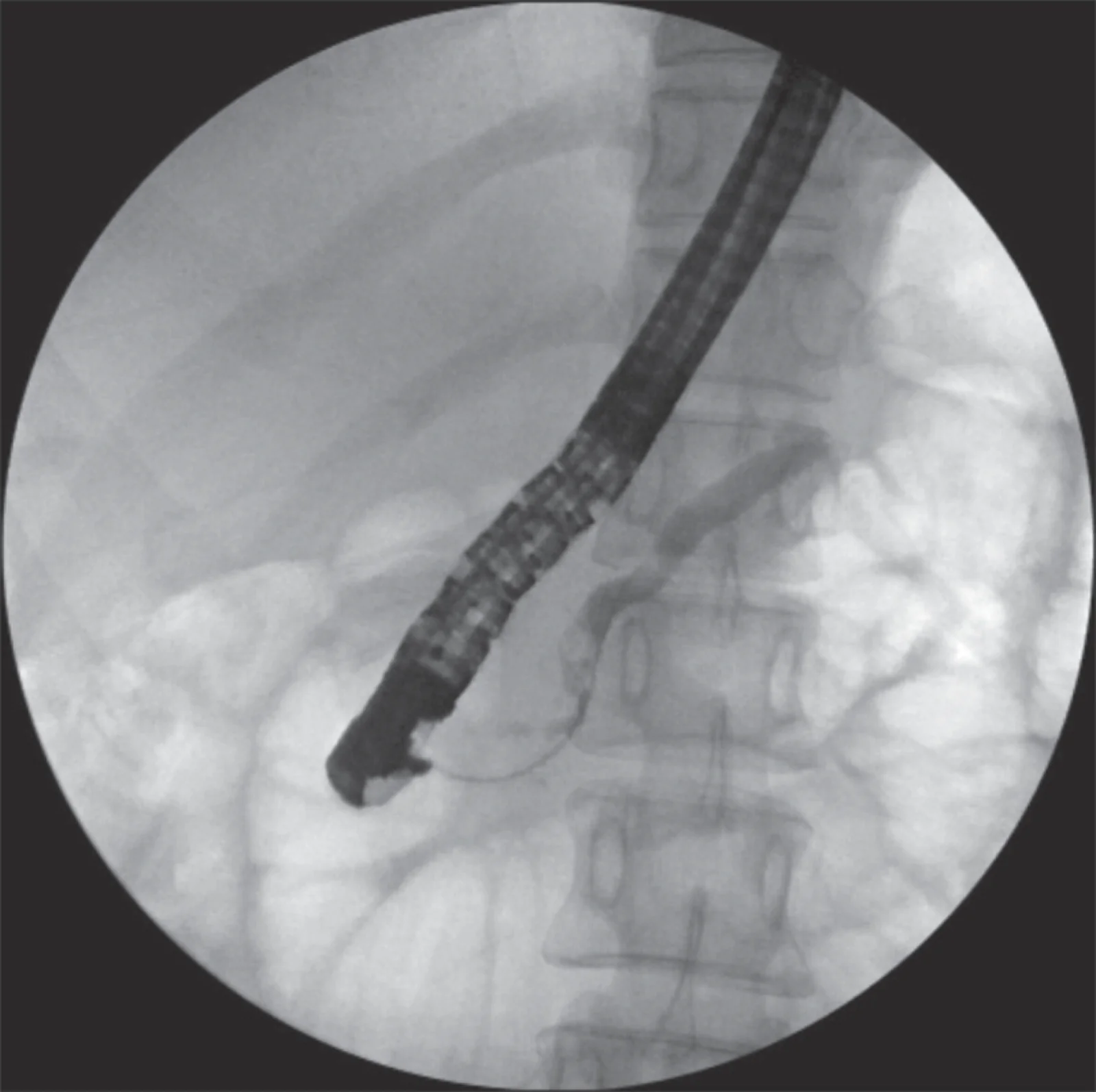
ERP demonstrating a main pancreatic duct stone with an associated stricture on the papillary side.

**Video 1** Temporary placement of a fully covered self-expanding metal stent to facilitate pancreatic duct stone extraction during a subsequent procedure.


**Fig. 2:**
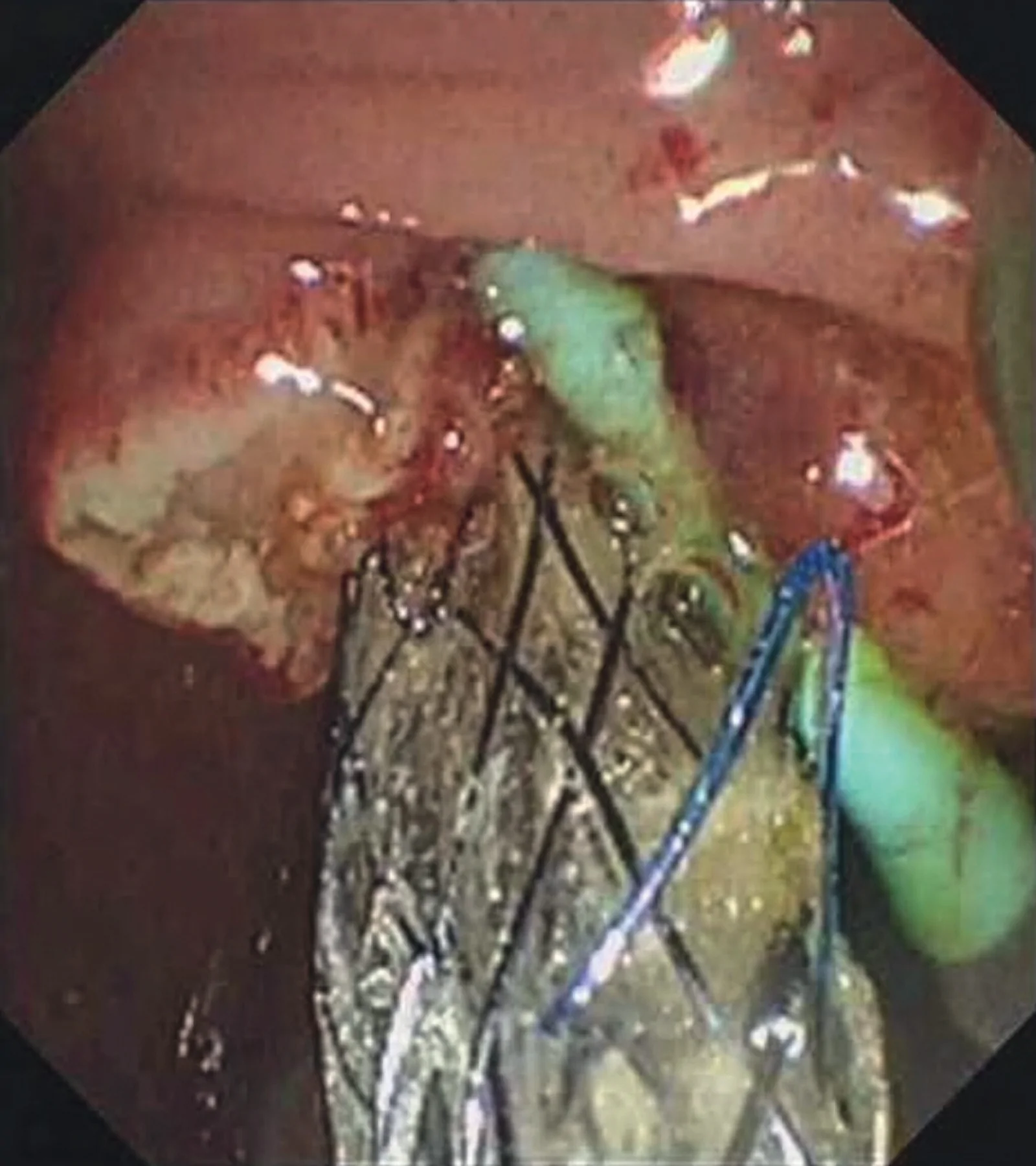
A 10×60-mm FCSEMS across the pancreatic duct stricture and a prophylactically placed 7-Fr×7-cm biliary plastic stent.

At repeat ERCP 41 days later, both stents were removed ( Fig. 3 ). An extraction balloon readily traversed the previously narrowed segment, and repeated balloon sweeps retrieved whitish stone fragments without lithotripsy ( Fig. 4 ). No immediate procedure-related adverse events occurred.

**Fig. 3:**
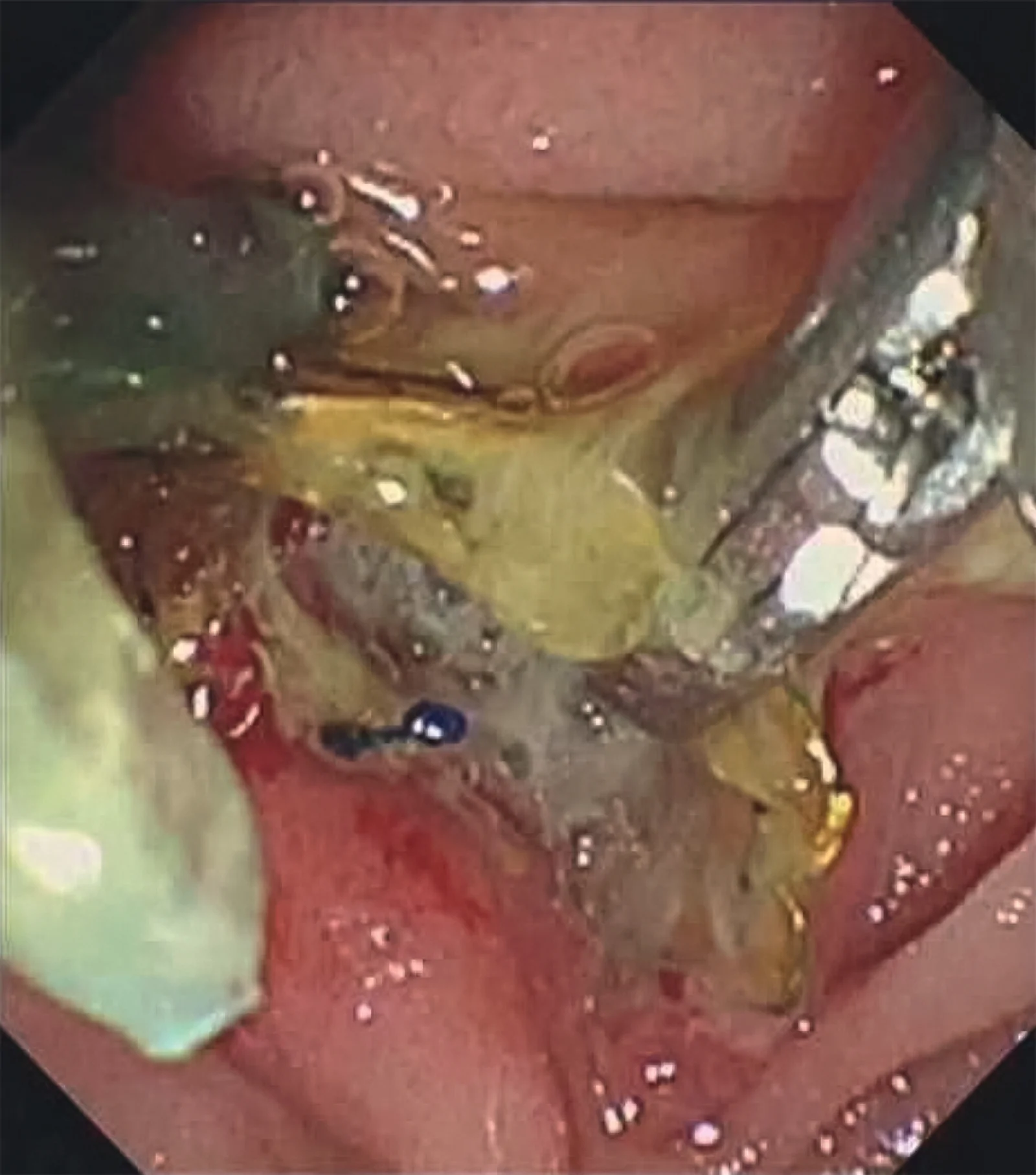
Removal of both stents using foreign-body forceps.

**Fig. 4:**
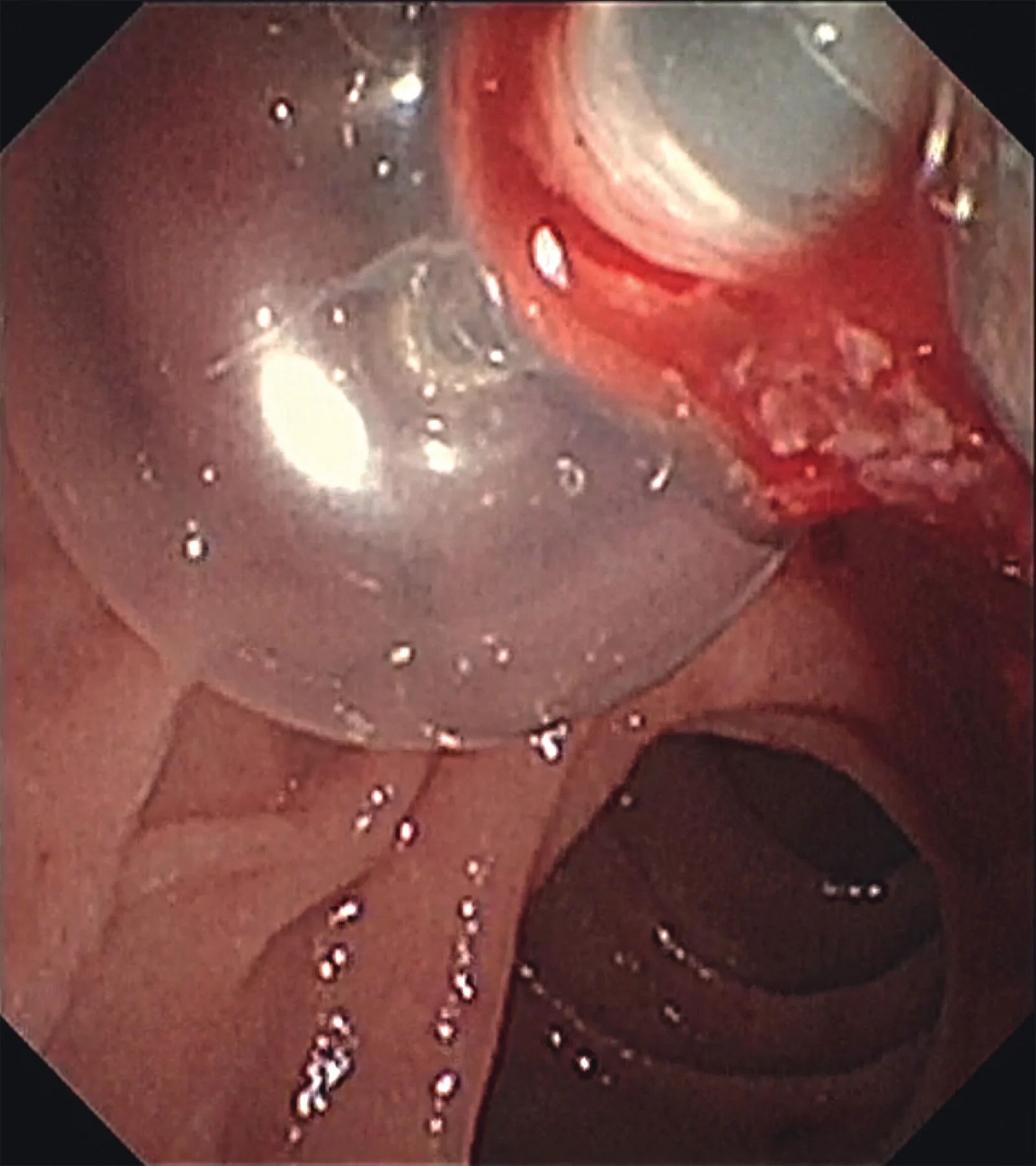
Balloon extraction of whitish, flocculent pancreatic duct stone fragments.

During 3 years of follow-up, the patient had no recurrent abdominal pain, pancreatitis, or related hospitalizations. For papillary-side strictures precluding routine stone retrieval, short-term intraductal FCSEMS placement may facilitate lithotripsy-free extraction and improve pancreatic duct drainage. 3​

Endoscopy_UCTN_Code_TTT_1AR_2AH

## Declaration of GenAI Use

During the preparation of this manuscript, the authors used ChatGPT, developed by OpenAI, solely for English-language editing to improve grammar, clarity, and readability. All artificial intelligence–assisted contents were reviewed and revised by the authors, who take full responsibility for the final manuscript.
